# EC-RPLIE: An Innovative Protocol for RPL in IIoT Networks

**DOI:** 10.3390/s26041371

**Published:** 2026-02-21

**Authors:** Mario A. Bonilla Brito, Daladier Jabba Molinares

**Affiliations:** System and Computer Engineering Department, Universidad del Norte, Barranquilla 080001, Colombia; mbonillaa@uninorte.edu.co

**Keywords:** IIoT, RPL, EBC, EC, IoT

## Abstract

The integration of Wireless Sensor Networks (WSNs) in Industrial Internet of Things (IIoT) applications presents significant challenges in terms of energy efficiency and network reliability, especially in dynamic industrial environments. The Routing Protocol for Low-Power and High-Loss Networks for Indoor Environments (RPLIE), while designed for low-power lossy networks (LLNs), lacks mechanisms to adequately balance energy consumption, a critical requirement for industrial sustainability. This research introduces an enhancement called Energy-Conscious Routing Protocol for Industrial Environments (EC-RPLIE), which incorporates the Expected Breakage Cost (EBC) metric to optimize energy distribution and network stability by managing medium-term jitter. Through extensive simulations in the Cooja environment, the performance of EC-RPLIE was evaluated against the state-of-the-art RPLIE across topologies of 11, 21, and 31 nodes. Quantitative results demonstrate that EC-RPLIE significantly reduces unnecessary retransmissions by maintaining a superior Packet Delivery Ratio (PDR) and optimizing parent selection. The protocol achieved energy savings of 9.6% in 11-node networks, which increased to 36.8% in high-density 31-node scenarios, effectively doubling the network persistence compared to RPLIE. Additionally, EC-RPLIE improved average latency by 12.68% in dense configurations, confirming its robustness in handling industrial traffic. These findings confirm that EC-RPLIE is particularly effective in high-density networks, where the EBC metric successfully mitigates the ‘retransmission storms’ typical of standard protocols. This proposal provides a robust framework for enhancing the sustainability and resilience of WSNs in Industry 4.0, offering a scalable solution that addresses the energy–reliability trade-off. The results lay the groundwork for future large-scale implementations in real-world industrial environments.

## 1. Introduction

Wireless Sensor Networks (WSNs) are foundational to the Industrial Internet of Things (IIoT), enabling the monitoring and control of complex industrial processes. Despite their versatility, WSNs face critical challenges regarding energy efficiency and reliability, primarily due to the limited battery capacity of nodes and the dynamic nature of industrial environments. Medium-term fluctuations in these settings often lead to link instability, frequent outages, and constant parent changes—factors that trigger unnecessary retransmissions and accelerate energy depletion [1].

The Routing Protocol for Low Power and Loss Networks (RPL) was designed to address these resource constraints. However, standard RPL and its extensions, such as RPLIE [2], often struggle to maintain optimal energy consumption in unstable scenarios. While RPLIE improves upon standard metrics, it does not fully mitigate the energy costs associated with maintaining a stable topology under fluctuating conditions [3]. To bridge this gap, the Expected Breakage Cost (EBC) metric is introduced. EBC provides a mathematical framework to evaluate the energy impact of link breakages and parent changes, identifying patterns across different link qualities—be they opportunistic, bad, or good.

This research proposes EC-RPLIE, an enhancement of the RPLIE protocol that integrates the EBC metric to optimize network sustainability. By accounting for the cost of link maintenance and the energy penalty of node re-parenting, EC-RPLIE aims to stabilize the network topology and extend its operational lifetime.

The performance of EC-RPLIE is evaluated through simulations in the Cooja environment across varying node densities (11, 21, and 31 nodes). This study analyzes key performance indicators, including the Expected Number of Transmissions (ETX), latency, and energy consumption. The results demonstrate that EC-RPLIE effectively reduces unnecessary overhead and enhances network longevity, particularly in high-density scenarios, providing a robust solution for resilient and sustainable IIoT deployments.

### Problem Definition and Research Objectives

Despite the evolution of the RPL protocol, a significant gap persists in how IIoT networks handle medium-term fluctuations. Current implementations often suffer from “parent-node instability,” where minor variations in link quality trigger frequent and costly topological changes. These changes generate a high Expected Breakage Cost (EBC)—a combination of signaling overhead and energy waste that standard metrics like ETX fail to quantify accurately. Consequently, there is no clear framework within RPLIE to distinguish between temporary interference and permanent link failure, leading to premature battery depletion in dense industrial deployments.

To address this, the primary objective of this research is to integrate the EBC metric into the RPLIE architecture to create EC-RPLIE. This study specifically aims to:

- Quantify the energy impact of parent switching and link maintenance in scenarios with varying node densities.
- Establish energy efficiency patterns by evaluating EBC (*k*, *p*) across different link categories (opportunistic, bad, and good).
- Validate the scalability of EC-RPLIE through Cooja simulations, measuring its ability to extend network longevity compared to the baseline RPLIE [2] protocol.

By defining these objectives, this work seeks to provide a definitive solution for creating sustainable IIoT infrastructures where energy conservation is prioritized alongside communication reliability.

## 2. Contextual Review

During this stage of the research, a comprehensive analysis of the Industrial Internet of Things (IIoT) paradigm was carried out, focusing on understanding its impact on industrial applications, its technological foundations, and its recent evolution. This analysis included the study of the IIoT architecture, which integrates intelligent devices, cyber-physical systems, and advanced communication technologies to optimize processes in sectors such as manufacturing, logistics, and energy.

The review focused on identifying the key technologies involved, such as intelligent sensors, communication protocols for LLN (Low-power and Lossy Networks), and data processing platforms. It also addressed the challenges associated with the implementation of these technologies, such as interoperability, energy efficiency, and secure data management.

### 2.1. Sensor Networks and Their Role in the Expansion of the Internet of Things

The Internet of Things (IoT) has become an essential component of modern life, aimed at improving our quality of life by connecting a multitude of smart devices, technologies, and applications that simplify our daily activities [4]. This interconnected ecosystem not only optimizes daily tasks, but also increases efficiency in various areas of our lives [4]. Within this context, the concept of Wireless Sensor Networks (WSNs) arises, which encompasses devices with specific characteristics, roles, and limitations, designed to collect, transmit, and analyze data.

**Figure 1:**
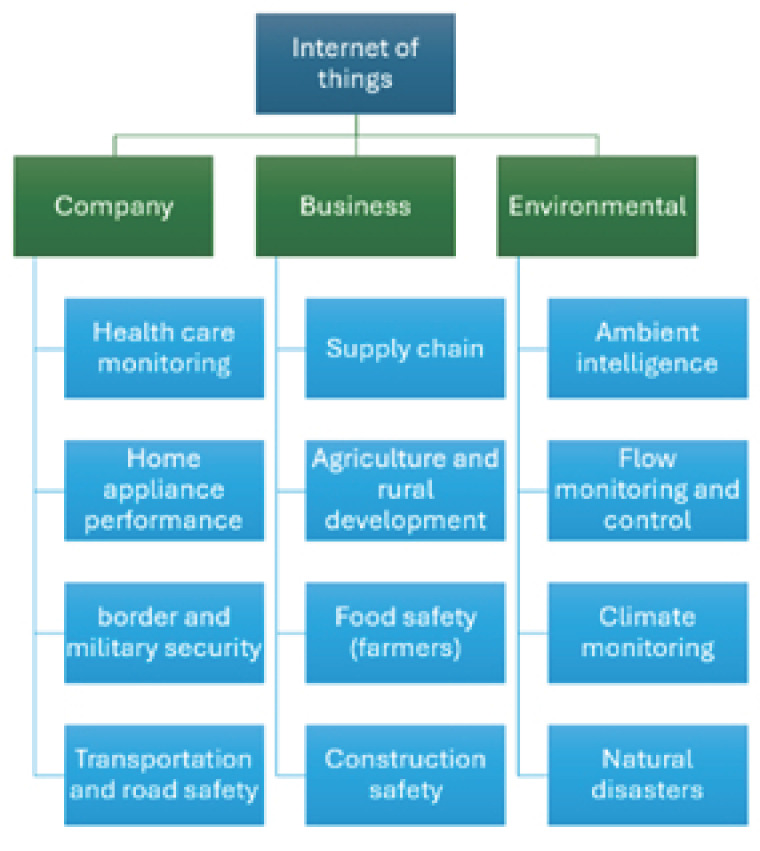
Classification of Internet of Things (IoT) applications. Source: Own elaboration.

Wireless sensor networks have gained relevance due to their ability to remotely monitor various scenarios [5]. These networks are used to detect behaviors of specific variables to make informed decisions, prevent potential disasters, or remotely control objects to perform specific actions [5]. In the agricultural sector, for example, WSNs have optimized irrigation systems, maximizing water use and improving crop yields [5]. In healthcare, they have revolutionized medical care by providing real-time access to critical patient indicators, enabling early detection of potentially life-threatening anomalies [5]. The diverse classification of these Internet of Things (IoT) applications is shown in Figure 1.

In addition, WSN applications extend to fields such as geology, where they are employed to monitor challenging environments such as volcanoes, rivers, and forests [4]. However, these networks face significant limitations, including low data processing capacity, which is directly related to the battery level of the sensors. As the processing load increases, so does the power consumption, forcing the design of sensors focused on capturing data and transmitting it to a receiver for processing, storage, forwarding, or alerting [4].

This technology enables real-time monitoring systems, where devices play different roles in the network, such as:

Data sources: in charge of measuring and communicating relevant information.

Data receivers: interested in receiving the information collected by WSNs.

Actuators: These are the fundamental elements for interaction with the physical environment. Actuators are devices that control actions based on received data, often functioning as data receivers [4]. In this scenario, Wireless Sensor Networks (WSNs) are crucial, as the remote control and monitoring of objects rely heavily on the continuous data collection provided by sensors for their correct operation [4].

### 2.2. Architectural Strategies for the Internet of Things

**Figure 2:**
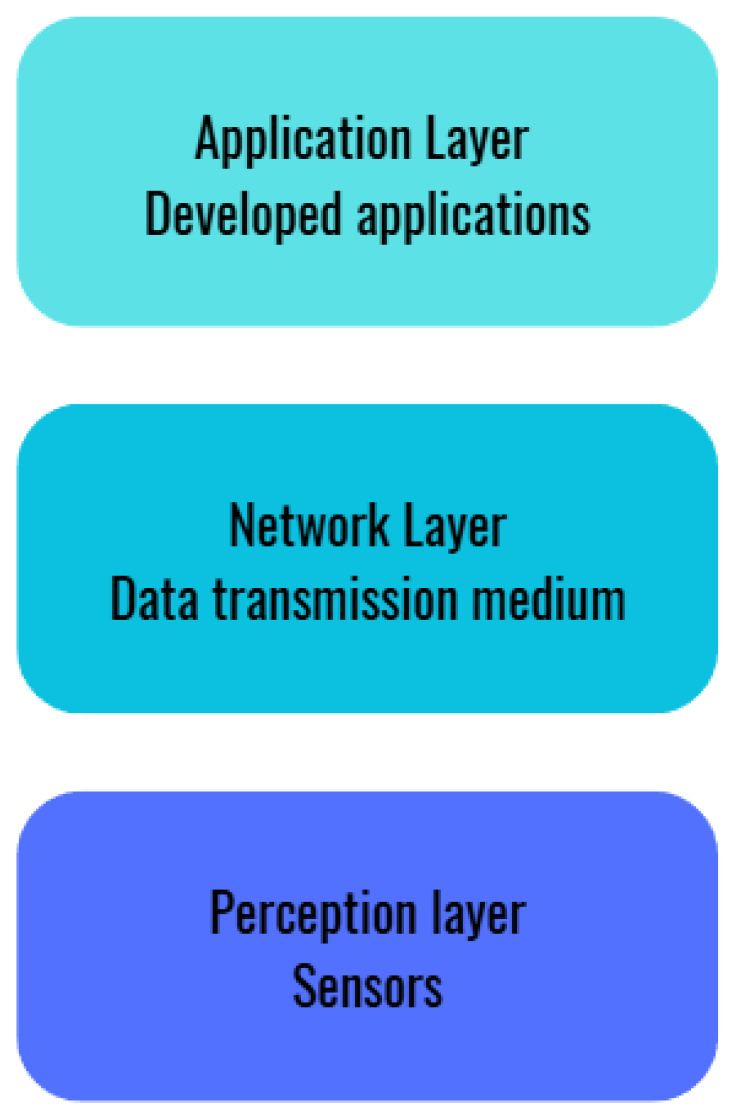
IoT architecture. Source: Own elaboration.

To connect billions of heterogeneous objects over the Internet, the IoT framework demands a flexible and scalable layered architecture. This structure allows any device connected to the network to be accessed and controlled remotely. To manage this complexity, IoT-based architectures are generally organized into three main functional layers: sensing, network, and application, as illustrated in Figure 2.

Perception layer:

This layer includes the physical sensors responsible for collecting and processing data from the environment. The sensors and actuators integrated in this layer perform functions such as measuring location, temperature, weight, movement, vibration, acceleration, and humidity, among other parameters. Interoperability in this layer is essential and requires standardized plug-and-play configuration mechanisms to integrate heterogeneous applications [4].

Network layer:

It comprises different types of networks (wired, wireless, private and public) that facilitate the transmission and processing of data collected at the sensing layer. This layer ensures that data is delivered efficiently and securely to its destination, acting as the backbone of connectivity in the IoT ecosystem [4,6].

Application layer:

User interfaces and services tailored to specific needs are developed in this layer. Notable applications include intelligent transportation systems, environmental monitoring, and remote medical systems. These applications exemplify how data processed in the lower layers can be transformed into useful services to improve people’s lives and optimize various processes [5,7].

### 2.3. What 5G Can Offer in IIot

The exponential increase in the use of wireless data and the proliferation of smart devices have catalyzed the development of fifth generation (5G) cellular technology, which, together with wireless sensor networks (WSNs), stands out in the Internet of Things (IoT) arena by providing reliable connectivity, low latency transmission, and efficient data processing. These features are essential in industrial automation and control systems, where efficiency and productivity depend on real-time transmission [4,8,9]. WSNs, employed to monitor remote environments such as hard-to-reach geological areas, face limitations in processing capacity, which are addressed by data redundancy and adaptability to node loss, while 5G uses advanced coding and error correction techniques to ensure fast and stable connections [9,10,11].

**Figure 3:**
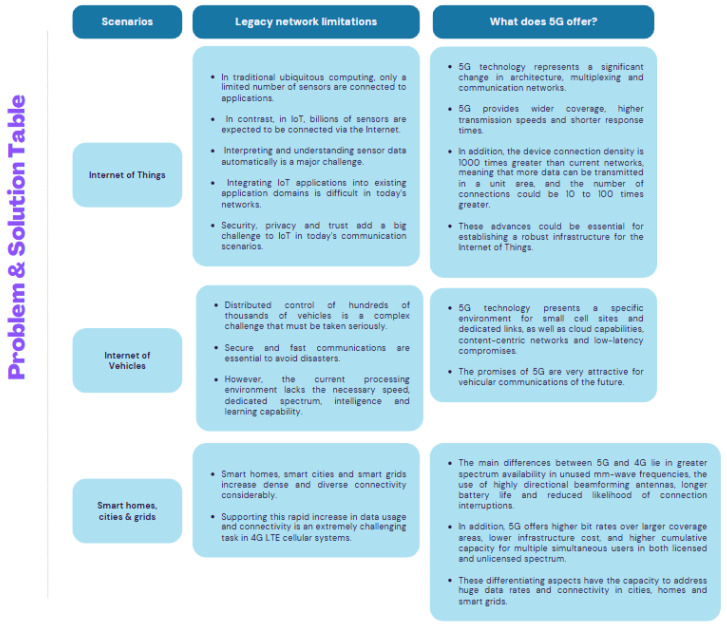
IIoT limitations that 5G can improve. Source: [6,8,9,14,15,16,17].

The integration of WSN with 5G boosts metrics such as latency, reliability, and security, essential for real-time applications and industrial automation. In the case of 5G, minimizing control plane traffic and optimizing network performance is crucial to avoid congestion and ensure reliable communications, even under high demand [12,13]. For their part, WSNs play a key role in the scalable architecture of IoT, interconnecting heterogeneous objects for remote monitoring and automation. These innovations make 5G and WSNs key enablers of advanced technology solutions, from intelligent transportation systems to industrial monitoring, while addressing challenges such as energy optimization and battery management [9,10,11,13,14]. The specific IIoT limitations that are improved through 5G integration are summarized in Figure 3.

### 2.4. Lln and 6LoWPAN: Routing Metrics and Protocols

**Figure 4:**
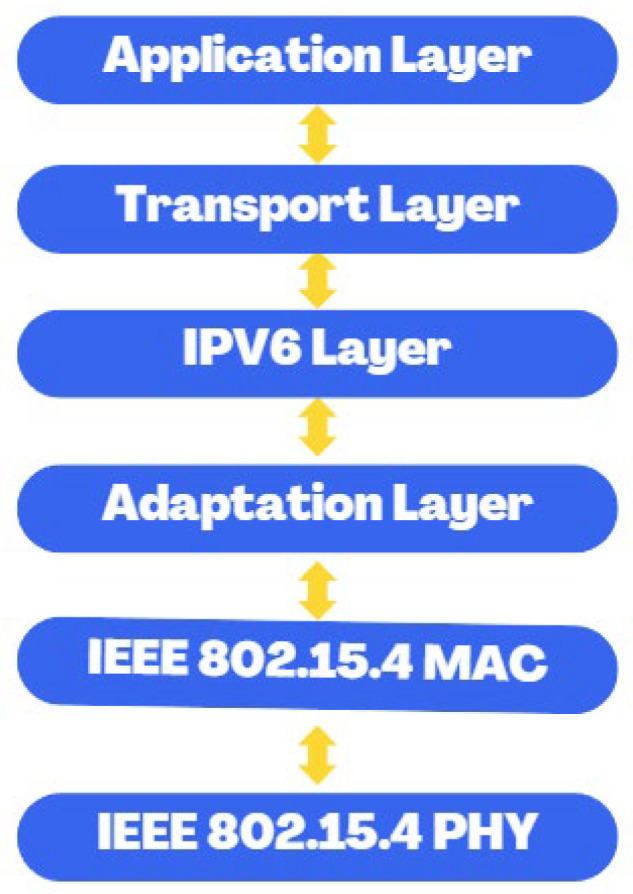
Model 6LoWPAN. Source: Own elaboration.

Low power and loss networks (LLNs) face significant challenges related to energy optimization, battery lifetime, and congestion reduction in the network, which requires the use of metrics to define optimal routes for data transmission, considering static and dynamic parameters of nodes and links [18]. Among these metrics stand out the number of hops, energy consumption, latency, expected number of transmissions (ETX), and throughput, which are used by the objective function (OF) to calculate Rank, a value that determines the distance from a node to the root node in directed acyclic destination-oriented directed networks (DODAG) and guides the selection of parent nodes in routing [19]. This approach is adopted by the Routing Protocol for Low-Power and Lossy Networks (RPL), which optimizes routes in LLNs, maximizing energy efficiency and ensuring reliable connectivity, making it a key solution in IoT environments and wireless sensor networks [18]. In addition, 6LoWPAN complements LLNs by offering efficient IPv6 connectivity through header compression and fragmentation to conform packets to the IEEE 802.15.4 standard, as shown in the 6LoWPAN model in Figure 4. Optimizing energy consumption and prolonging the lifetime of nodes and batteries. This protocol is highly flexible and scalable, supporting meshed and hierarchical configurations to cover large areas with redundancy in case of failures, and integrates effectively with RPL to select optimal routes in high-loss, low-power networks using adaptive metrics that improve overall performance [18,19].

**Figure 5:**
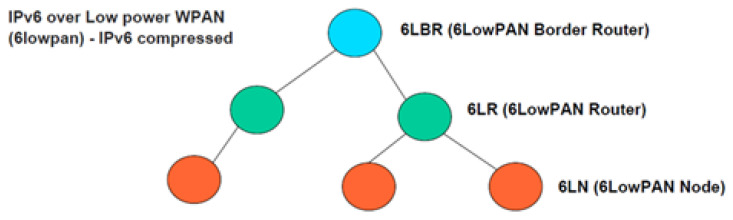
Network topology for IPv6 over Low Power Wireless Personal Area Networks (6LoWPAN). Source: [20].

In LLNs, routing efficiency is based on fundamental metrics that allow determining optimal routes for data transmission. These metrics include the number of hops, which measures the intermediate nodes on a route; energy consumption, which evaluates the energy cost to extend the lifetime of the nodes; latency, which reflects the time required to transmit a packet from the source to the destination; the expected number of transmissions (ETX), which analyzes the quality of the links when considering the required retransmissions; and throughput, which measures the network’s capacity to handle data. These metrics are integrated into an objective function (OF), which calculates a value called Rank, representing the logical distance from a node to the root node within DODAG (Destination-Oriented Directed Acyclic Graph) structures, guiding the selection of parent nodes to optimize routing [18,19]. The organization of these nodes and the resulting network topology for 6LoWPAN are illustrated in Figure 5.

### 2.5. IIot (Industrial Internet of Things): Challenges, Protocols, and Solutions

The Industrial Internet of Things (IIoT) has revolutionized industrial processes by connecting smart devices, sensors, and systems, improving operational efficiency, reducing costs, and anticipating equipment failures. Sectors such as manufacturing, energy, transportation and healthcare have adopted IIoT to leverage real-time data. However, its implementation comes with technical and operational challenges, such as selecting efficient communication protocols, ensuring security against cyber-attacks, and the ability to integrate between diverse devices and systems [21,22].

Protocols such as RPL and 6LoWPAN play a crucial role in IIoT networks by managing efficient data transmission in resource-constrained devices. RPL uses metrics such as power consumption and latency to select optimal routes in tree topologies, while 6LoWPAN allows these devices to connect via IPv6, integrating them into global networks. In addition, scalability is a challenge in industrial networks, as they require handling large volumes of data. Technologies such as 5G and Edge Computing are essential to improve the capacity of IIoT networks, ensuring high-speed, low-latency connectivity in environments with thousands of devices [21].

**Figure 6:**
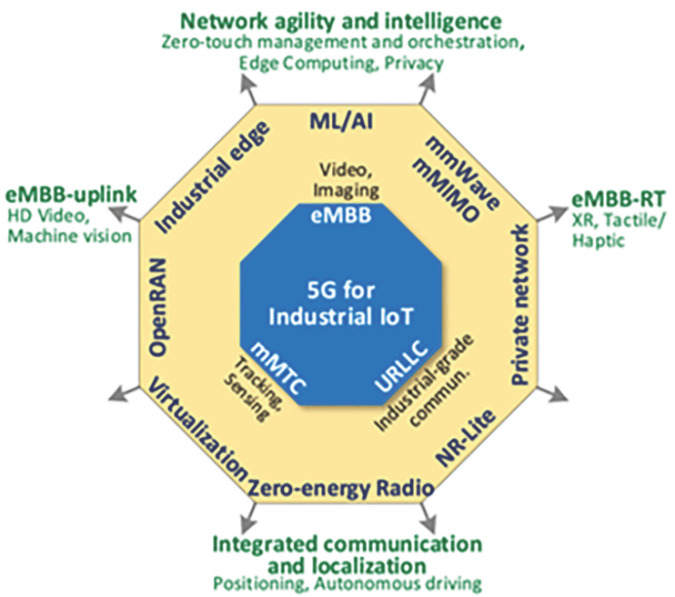
Beyond-5G vision, 5G architecture and design trends for IIoT. Source: [22].

Security and interoperability are critical aspects in IIoT. The adoption of blockchain, artificial intelligence, and open standards such as OPC UA improves protection against cyber-attacks and facilitates communication between devices from different manufacturers. Looking ahead, IIoT points towards increased automation with artificial intelligence and machine learning, optimizing industrial networks, and promoting sustainability through integration with emerging technologies such as quantum and cloud computing [22]. This forward-looking perspective, including the Beyond-5G vision and current design trends for IIoT, is represented in Figure 6.

### 2.6. RPL Routing Protocol

**Figure 7:**
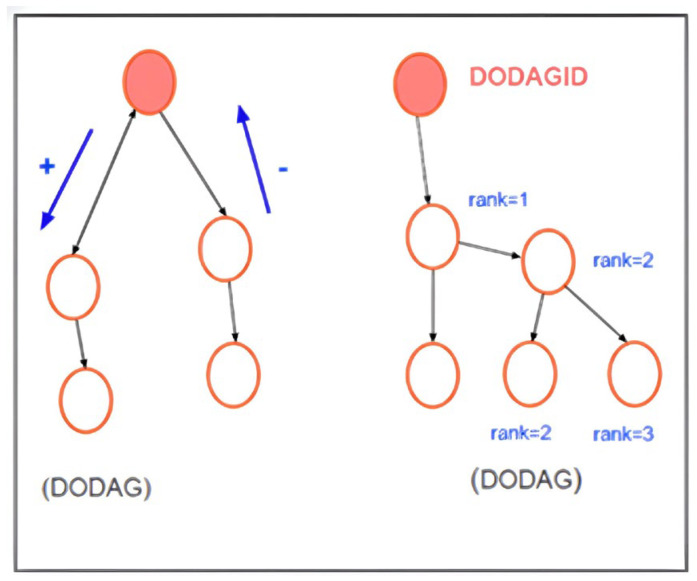
RPL topology. Source: [23].

The Routing Protocol for Low-Power and Lossy Networks (RPL) is a solution designed to improve connectivity in IoT networks by building efficient hierarchical topologies that optimize the use of limited resources, such as energy and processing. This protocol uses a structure called DODAG (Directed Acyclic Graph oriented to a destination), in which nodes are hierarchically organized towards a root node, as illustrated in the RPL topology in Figure 7. The process of building a DODAG begins with the emission of DIO (DODAG Information Object) messages by the root node, which contain key information for the child nodes to calculate their position (Rank) and select optimal routes based on metrics such as latency and energy consumption. Additionally, nodes can exchange DAO (Destination Advertisement Object) messages to advertise available routes or DIS (DODAG Information Solicitation) messages to obtain details about the network, which allows a dynamic update of the topology to adapt to changes in the network [19,23].

RPL operates in two main modes: non-storage mode, in which all routing information resides at the root node, and storage mode, where intermediate nodes store routing information, allowing for more distributed routing. These modes have significant implications on network performance, especially in terms of memory consumption and processing. In addition, objective functions (OFs) play a crucial role in determining optimal communication routes. Among the most commonly used are OF0 and MRHOF (Minimum Rank with Hysteresis Objective Function), which are based on metrics such as hop count and expected transmission count (ETX). However, as networks grow in density, these functions can generate bottlenecks by introducing long hops, underscoring the importance of optimizing routing metrics [18,23].

Recent research has identified a comprehensive set of constraints and metrics, such as number of hops, energy, latency, and ETX, that can be integrated into the objective function to optimize routes in IoT networks. These advances are critical to address the limitations of the RPL protocol in dense networks. In particular, the comprehensive review presented in [22] highlights innovative solutions that improve efficiency and scalability by refining the metrics used in the objective functions. The paper also provides a comprehensive analysis of related research up to 2018, consolidating an essential resource for the development of future applications of the RPL protocol in IoT networks [17,22].

### 2.7. Related Work

The article [22] covers the metrics used in RPL and IIoT protocols as they relate to what 5G can bring to the industry, and provides guidance to researchers on current and future trends in measuring these metrics [23,24]. It is hoped that this work will contribute to the advancement in the development of latency–reliability solutions in RPL and IIoT with 5G protocol, and enable improved efficiency and effectiveness in the implementation of these protocols in the industry.

Specific studies have explored several methods to improve routing metrics and objective functions in RPL focused on correlated IIoT in indoor environments to adapt to different requirements in particular application contexts [23,24,25,26,27,28]. Some of these studies, such as WRF-RPL, utilize a composite routing metric to construct and maintain a network structure known as a DODAG, thereby balancing the traffic load within the network. This protocol also employs a random weighted packet retransmission scheme to improve packet delivery reliability and reduce energy consumption [25]. Another proposal is the Adaptation and implementation of the BF-ETX objective function in the RPL protocol, which improves QoS indicators such as network lifetime, packet delivery, and energy savings [25]. In [2], RPLIE outperforms the RPL standard in terms of packet delivery performance and overhead. Its new routing metric (EBC) considers the cost of link breakage, which allows a better evaluation of link quality. In [24], P2P-RPL presents a geographical collision avoidance approach to improve the performance of indoor multi-hop wireless networks. Authors in [26] introduce SC-RPL, displaying that the interaction between nodes in IIoT exhibits strong regularity or sociality, which can potentially improve the efficiency of IIoT. One satisfies the requirement of delay-sensitive information transmission, and the other focuses on load balancing of social and cognitive IIoT networks. In [27], SA-RPL focuses on the problem of load balancing and energy efficiency in wireless sensor networks (WSNs) using the RPL routing protocol and the TSCH access method of the IEEE 802.15.4e standard. SA-RPL distributes the energy consumption among the nodes closer to the destination node, resulting in a longer lifetime for the network as a whole.

### 2.8. Contiki OS

Contiki OS is an operating system designed for resource-constrained IoT (Internet of Things) devices, such as sensors and actuators in low-power, high-loss networks (LLNs), providing an efficient and modular platform for developing IoT applications. This system supports essential protocols such as 6LoWPAN, CoAP and RPL, in addition to providing native IP connectivity with support for IPv6, which facilitates integration into global networks. Its protothread-based programming model enables lightweight multitasking, combining the simplicity of cooperative multitasking with the benefits of pre-emptive multitasking, ideal for devices with restricted capabilities. Contiki also includes the Cooja simulator, widely used to test applications and simulate IoT networks before their implementation in hardware, making it a key tool for research and development in this field. In terms of energy efficiency, it introduces mechanisms such as the ContikiMAC protocol, which optimizes energy consumption through radio duty cycling techniques. In addition, its flexibility and compatibility with low-cost hardware have made it a preferred choice for large-scale IoT projects, such as environmental monitoring, industrial automation, and smart homes [22].

## 3. EC-RPLIE: A Novel Protocol for RPL in IIoT Networks

Link dynamics in wireless networks, derived from environmental and structural changes, represent a critical challenge in the management of communication protocols, particularly in Low Power and Loss (LLN) wireless networks. These changes, characteristic of industrial environments, directly affect the performance of IIoT (Industrial Internet of Things) networks. While previous research has addressed link dynamics over short- and long-term horizons, this study focuses on medium-term fluctuations, a relevant phenomenon in scenarios where variations occur in intervals of minutes to hours, such as the opening and closing of doors and windows in indoor environments.

In this context, we present EC-RPLIE, an enhancement of the RPLIE protocol that introduces the Expected Breakage Cost (EBC) metric as a key tool to assess and optimize energy efficiency in IIoT networks. This approach allows measuring the costs associated with link breakages and parent node changes, critical factors in the stability of industrial networks. EC-RPLIE not only addresses the limitations of the RPLIE protocol, traditionally designed for WSN LLNs, but also prioritizes efficient energy consumption, a crucial aspect for IIoT applications where energy sustainability is an inescapable requirement.

The analysis was performed through simulations in the Cooja environment, evaluating metrics such as ETX, transmit and listen duty cycles, and average time between packets. The results provide a comprehensive perspective on how the EC-RPLIE protocol improves medium-term jitter management, optimizing energy efficiency without compromising network performance. This approach proves to be especially effective in dynamic industrial networks, establishing a basis for implementation in real-world environments that demand energy-sustainable solutions.

### 3.1. EBC (Expected Cost of Breach) and Its Role

Expected Breakup Cost (EBC) is an important metric for assessing link reliability in Low-Power and Lossy Networks (LLNs), such as those using the Routing Protocol for Low-Power and Lossy Networks with Improved Efficiency (RPLIE). In these networks, nodes rely on wireless communication, which is susceptible to signal degradation due to factors such as distance, interference and obstacles. EBC takes into account several factors that affect link stability and power consumption, making it an essential component in protocols that prioritize energy efficiency and data reliability.

The formula for calculating *EBC* is:

$$EBC\;(k,p)=\frac{BC}{MT\left(k,p\right)\cdot TL(k)}$$

where:

- *BC* (Breakage Cost): Number of wasted transmissions.
- *MT* (Maintenance Time): Average time a node maintains a link.
- *TL* (Traffic Load): Node traffic load.
- The relationship (*k*, *p*) defines the link between a child and its parent.

ETX (Expected Transmission Count): A metric that estimates the number of transmissions required to successfully send a packet over a link, usually influenced by the packet loss rate.

Number of Failed Transmissions: The ratio of packets lost to packets received, reflecting the reliability of the link.

Maintenance Time (*MT*): The time required to maintain a stable link, based on the ETX and a scaling factor.

Traffic Load (*TL*): The traffic load on the network, inversely related to the time between packets, which influences the communication frequency.

Key variables of each node:

- Expected Transmission Count (ETX): Base metric of the RPL protocol.
- EBC (Expected Breakage Cost): Evaluation of the cost associated with link breaks.
- Duty cycles: Listening and transmission.
- Inter-packet time: Average time between transmitted packets.

#### 3.1.1. Classification of Links According to EBC

Links in RPLIE networks can be classified as good, bad, or opportunistic, according to their EBC values.

Bad Links:

They are characterized by frequent failures and high EBC. These links result in many retransmissions (high BC), which increases energy consumption and network instability.

Impact: Increased transmission attempts due to link instability, increased power consumption, and possible delays in data delivery.

Good Links:

Stable links with low EBC, indicating fewer retransmissions and consistent data delivery.

Impact: These links help achieve efficient energy usage and lower latency in data transmission.

Opportunistic Links:

These links exhibit mixed behavior, being unstable at times, but offering high transmission efficiency when conditions are favorable. Their EBC is generally intermediate.

Impact: Although they offer short-term performance benefits, they can generate an increase in EBC during periods of instability, which must be carefully managed.

#### 3.1.2. EBC Energy Impact

EBC directly influences energy consumption in wireless networks. High EBC values imply frequent retransmissions, which increases energy expenditure, as nodes activate their radio for communication more frequently. Conversely, low EBC values indicate fewer retransmissions and thus lower energy consumption.

High EBC (Bad Links): More retransmissions lead to higher power consumption due to frequent use of the communication hardware.

Low EBC (Good Links): Stable links reduce the number of retransmissions and energy usage.

Intermediate EBC (Opportunistic Links): These links may offer energy savings during stable phases, but consume more energy during unstable phases due to retransmissions.

#### 3.1.3. Challenges in EBC-Based Routing Protocols

Although EBC provides a robust method for selecting reliable and energy efficient routes, there are several challenges that need to be addressed:

Dynamic Link Conditions: EBC values fluctuate as network conditions change, such as node mobility, interference, or environmental factors. The protocol must adapt quickly to these changes to avoid unnecessary energy consumption.

Trade-off between Energy Efficiency and Stability: RPLIE seeks to balance network stability with the need for low energy consumption, which requires careful management of EBC and ETX metrics to optimize performance without compromising reliability.

### 3.2. RPL and Mrhof (Minimum Rating Target with Hysteresis)

RPL uses several objective functions to optimize routing decisions. The MRHOF (Minimum Rank with Hysteresis Objective Function) [16], commonly used in RPL, selects routes based on the lowest cost, usually measured by metrics such as ETX and EBC.

MRHOF objective: Minimize the total cost of routing, including retransmissions (related to energy consumption) and route stability. Use hysteresis to avoid frequent route changes that can destabilize the network.

Role of EBC in MRHOF: In RPLIE, the EBC metric is incorporated in MRHOF [9] to evaluate the stability of links over time. Links with lower EBC are prioritized because they offer more stable routes and lower energy consumption.

### 3.3. Novel Protocol Design

**Figure 8:**
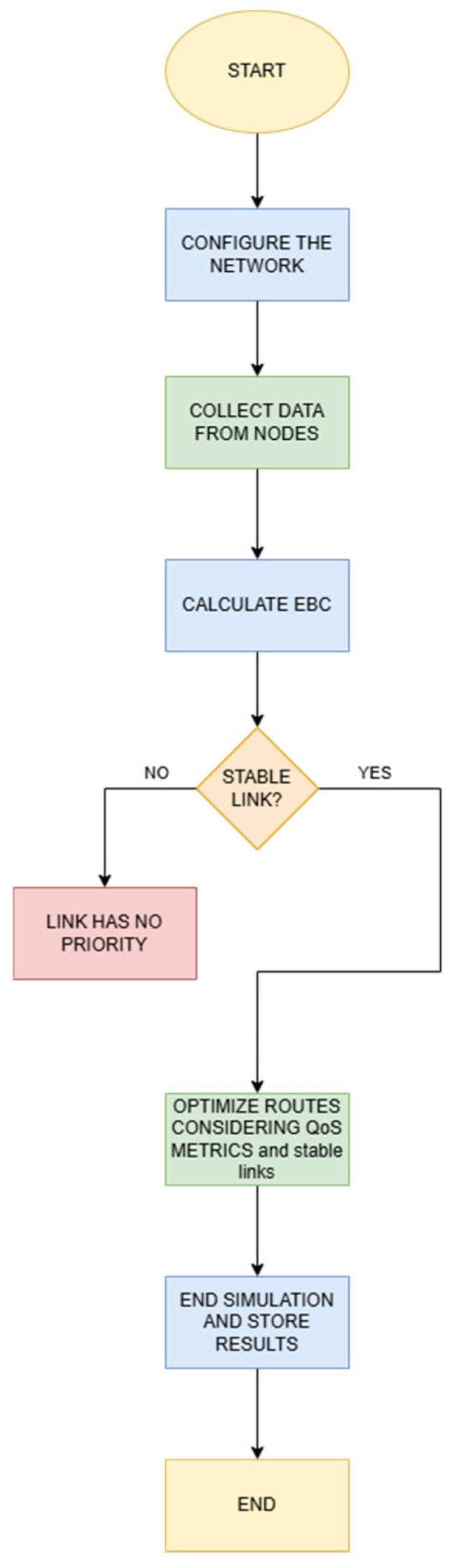
Diagram of the proposed EC-RPLIE Protocol.

The flowchart for the proposed EC-RPLIE protocol, illustrated in Figure 8, introduces a dynamic routing methodology designed specifically for the hostile conditions of the IIoT. Unlike the RPL standard, which is usually static, this protocol implements a feedback loop where data collection from the nodes feeds directly into the Energy Balanced Characteristic (EBC) calculation algorithm. This stage is essential, as it allows the protocol to evaluate not only the efficiency of the path, but also the energy health of each device, achieving a load distribution that prevents premature depletion of strategic nodes in the industrial plant.

The most relevant technical innovation is seen in the decision fork based on link stability. In an industrial environment, the presence of motors, metal structures, and electromagnetic noise generates intermittent links; EC-RPLIE addresses this challenge by actively discarding low-priority routes that do not meet stability thresholds. By integrating Quality of Service (QoS) metrics into route optimization, the protocol ensures that critical data traffic is routed only through validated links, thus optimizing energy consumption by minimizing unnecessary retransmissions and maximizing network reliability under stressful conditions.

#### 3.3.1. Configuration of a New Simulation

The user must open Cooja and select the “File -> New Simulation” option. This will open a dialog box that will allow the user to configure the initial parameters of the simulation.

**Figure 9:**
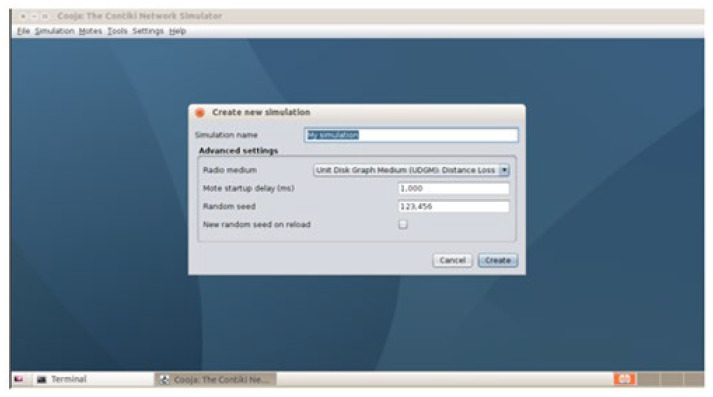
Radio medium. Source: Own elaboration.

**Figure 10:**
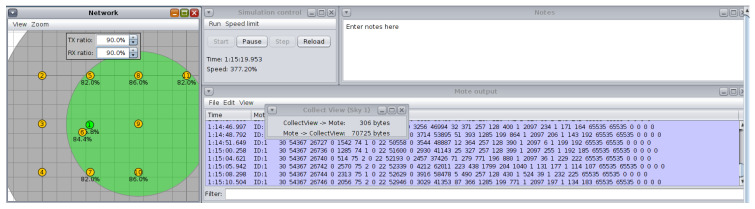
Node selection. Source: Own elaboration.

**Figure 11:**
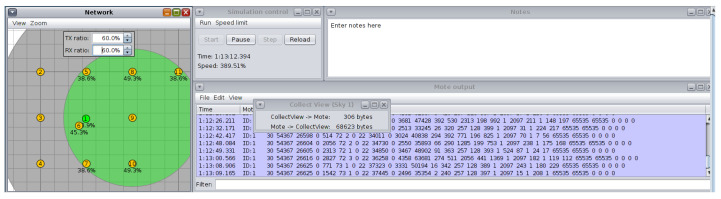
Varying Rx ratio and Tx ratio. Source: Own elaboration.

The test scenarios depicted in Figure 9, Figure 10 and Figure 11 were run using the Cooja simulator. During each run, a log file was generated containing a detailed record of the data produced by the motes, in addition to the configuration files in XML format required for the simulation. Each scenario was carefully configured to capture key information related to network behavior and speckle performance.

From each of these simulations, analyses related to network lifetime, energy consumption, PDR, and latency were performed.

#### 3.3.2. Assigning a Name and Selecting the Network Type

In the field intended for the simulation name (Simulation name), a name representative of the scenario to be simulated must be provided.

In the Radio Medium section, it is important to choose a network model suitable for the characteristics of the protocol to be developed. For example, for a typical IoT (Internet of Things) environment, it is recommended to select the UDGM: Distance Loss model, since it simulates IEEE 802.15.4 networks with low power consumption and short distance transmission, ideal characteristics for low power and long-range networks (LLNs).

#### 3.3.3. Selection of Sink and Sender Nodes

At this stage, it is necessary to choose the nodes that will act as the sink (central receiver) and the nodes that will send data (senders). The sink will function as the collection point for all information, while the senders will be responsible for transmitting the packets. This selection should be made based on the purpose of the simulation, ensuring an adequate distribution of roles in the network.

#### 3.3.4. Rx Ratio and Tx Ratio Fluctuations Configuration

To simulate link fluctuations, periodic adjustment of the values of the receive (Rx ratio) and transmit (Tx ratio) rates is required. These changes represent the dynamic variations that can occur in real environments due to factors such as the opening and closing of doors or windows. The simulation should be programmed so that these variations occur at specific intervals, allowing the behavior of the protocol under changing conditions to be evaluated. Variations were made for 1 h every 5 min from Tx: 90% Rx: 90% to Tx: 60% and Rx: 60%.

#### 3.3.5. Data Collection with Collect View

**Figure 12:**
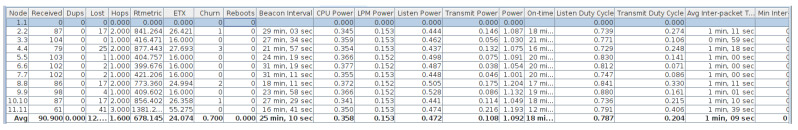
Information on each of the network nodes. Source: Own elaboration.

During the simulation, the Collect View tool is used to collect detailed information from each node, as shown in Figure 12. This includes metrics related to packet reception and transmission, energy consumption, and connectivity status. This data allows for a comprehensive analysis of network performance and validation of the impact of configured jitter.

#### 3.3.6. Calculation of the EBC Metric

Once the individual node data has been collected with Collect View, the EBC (Expected Break Cost) metric is calculated. This metric evaluates the expected cost of interruptions in the links, providing a quantitative measure of the impact of fluctuations. The calculation is performed using the information obtained from each node, such as transmission and reception rates, to determine the costs associated with temporary disconnections.

#### 3.3.7. Network Lifetime, PDR and Energy Consumption Analysis

Using Collect View data, calculations are performed for:

Network lifetime: the total operating time until the network ceases to be operational due to energy depletion of critical nodes is calculated.

PDR (Packet Delivery Ratio): the ratio between packets sent and received is measured to evaluate the efficiency of data delivery.

Overall energy consumption: the total energy consumed by the network during the simulation period is estimated, providing an overview of the overall energy performance.

These calculations allow comparing different configurations for 11, 21, and 31 nodes and evaluating the effectiveness of the designed protocol against the simulated fluctuating conditions.

### 3.4. Simulation Scenarios

**Figure 13:**
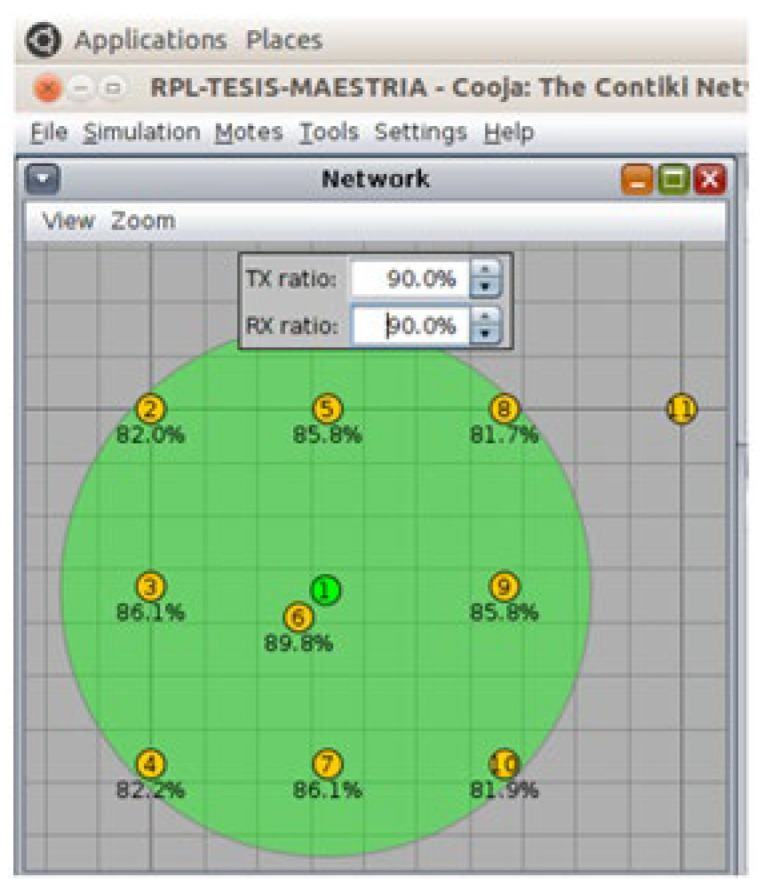
Scenario with 11 nodes. Source: Own elaboration.

**Figure 14:**
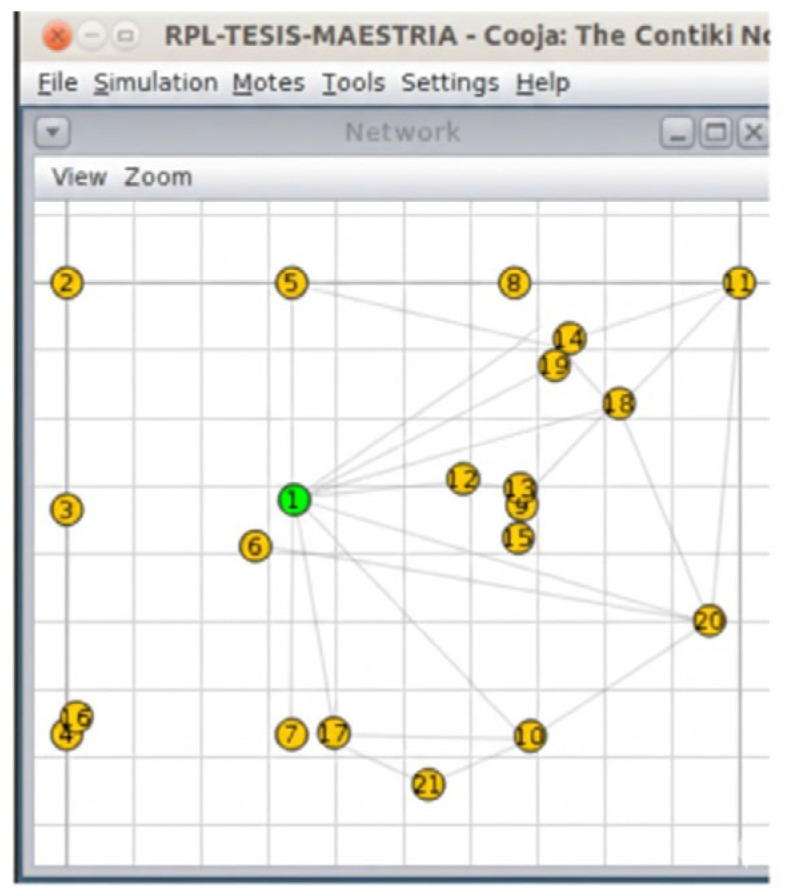
Scenario with 21 nodes. Source: Own elaboration.

**Figure 15:**
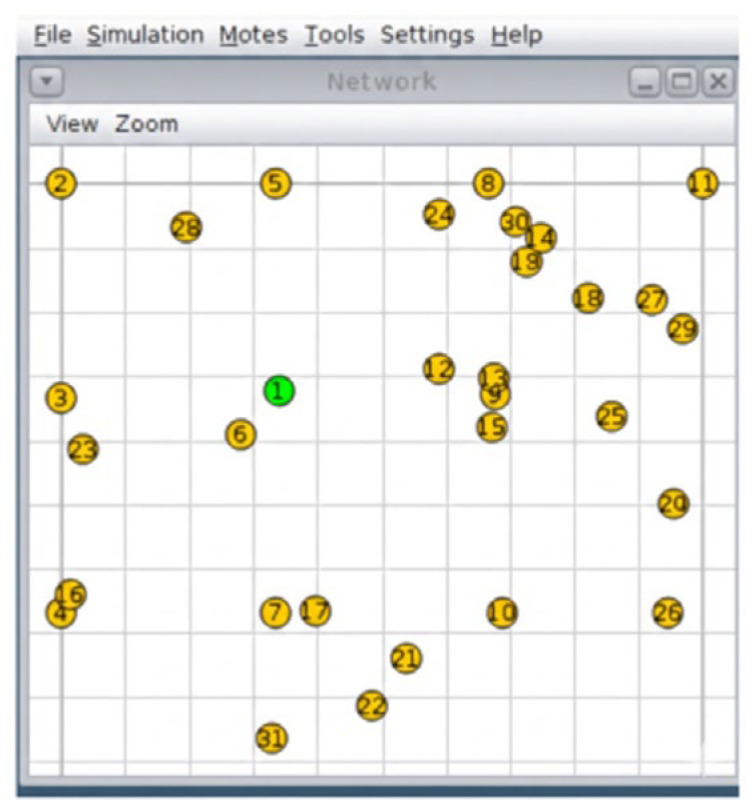
Scenario with 31 nodes. Source: Own elaboration.

Three network configurations were made, each with a different number of nodes, in order to compare and evaluate the effectiveness of the designed protocol against the simulated fluctuating conditions and thus improve the energy consumption of the network. These configurations consist of scenarios with 11, 21, and 31 nodes, as illustrated in Figure 13, Figure 14, and Figure 15, respectively.

## 4. Data Review

To provide a comprehensive overview of the experimental phase, three distinct simulation scenarios were executed to evaluate the performance of the proposed EC-RPLIE protocol against the standard RPLIE. These simulations varied in network density to test scalability and robustness under different industrial conditions. The technical configurations and the consolidated quantitative results for metrics such as PDR, latency, energy consumption, and network lifetime are summarized in Table 1. This comparative summary serves as the baseline for the detailed analysis of each performance indicator presented in the following sections.

### 4.1. Network Lifetime

**Figure 16:**
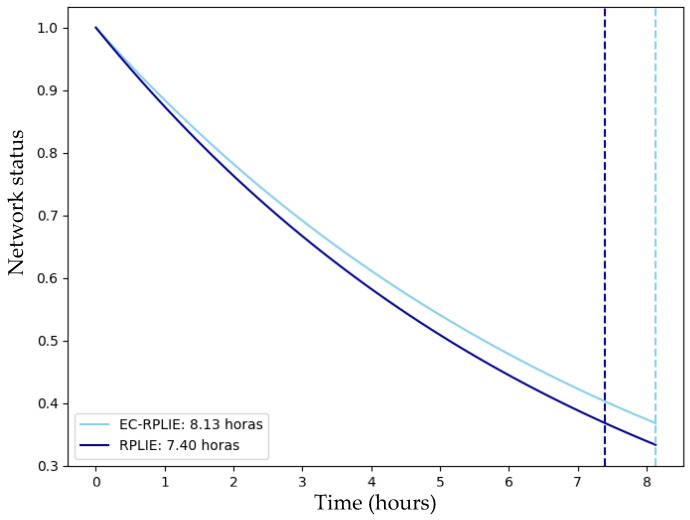
Lifetime comparison of the 11-node network. Source: Own elaboration.

**Figure 17:**
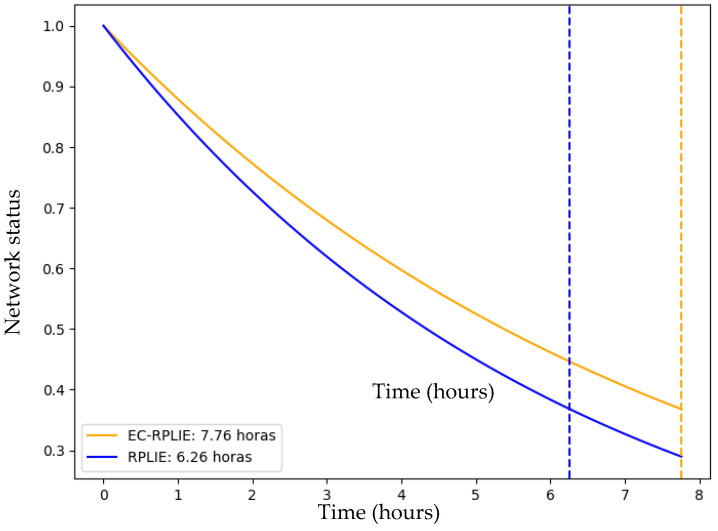
Lifetime comparison of the 21-node network. Source: Own elaboration.

The simulations conducted in Cooja for networks of 11, 21, and 31 nodes reveal a consistent performance trend, demonstrating that the EC-RPLIE protocol achieves superior energy efficiency and an extended operational lifetime compared to the standard RPLIE. In the 11-node scenario, EC-RPLIE maintained an average energy consumption of approximately 1085 Joules, significantly lower than the 1250 Joules averaged by RPLIE. This comparison of the operational lifetime for the 11-node network is illustrated in Figure 16. This efficiency remains evident as the network scales; in the 21-node configuration, although both protocols show increased consumption, EC-RPLIE consistently operates at lower energy levels, directly translating into a more sustained active state for the nodes, as compared in Figure 17.

**Figure 18:**
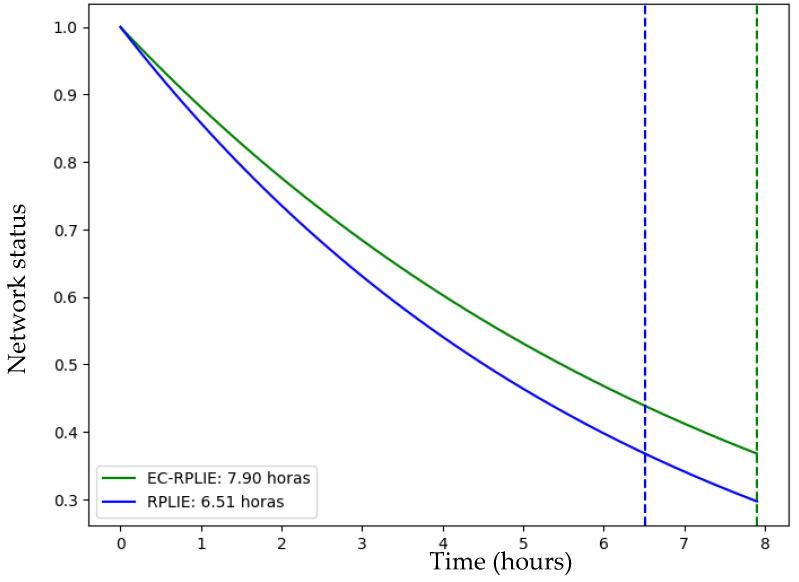
Lifetime comparison of the 31-node network. Source: Own elaboration.

The graphical representation of these states illustrates a transition from active (1) to inactive (0) as energy reserves are exhausted. Throughout the experiments, the dashed lines—which delineate the functional lifetime of each protocol—confirm that EC-RPLIE consistently delays the onset of node depletion. Even in the more complex 31-node network, where EC-RPLIE exhibits a more varied consumption range (1030 to 1936 Joules) compared to the more uniform but higher consumption pattern of RPLIE (up to 2200 Joules), the former maintains a clear advantage in longevity, which is detailed in Figure 18. Ultimately, the data indicates that EC-RPLIE optimizes resource distribution, effectively postponing network failure and enhancing overall robustness across all tested scales.

### 4.2. Energy Consumption

**Figure 19:**
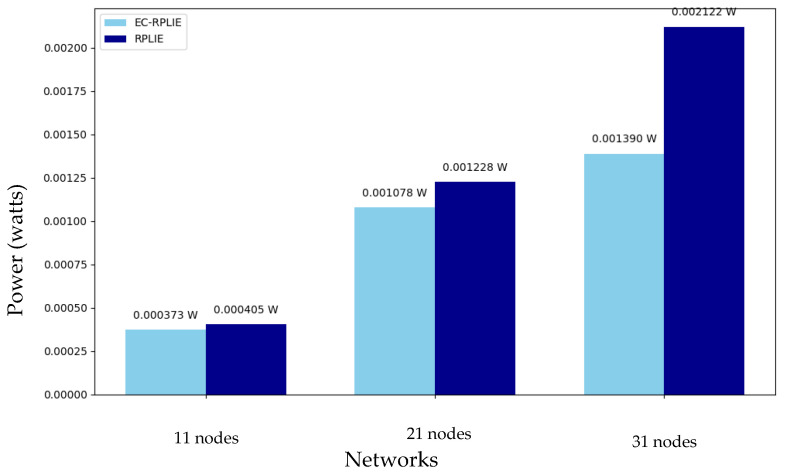
Power consumption between networks. Source: Own elaboration.

The experimental data across all tested scenarios confirms that EC-RPLIE systematically outperforms RPLIE in terms of energy conservation and operational efficiency. In the 11-node network, EC-RPLIE demonstrated a total energy expenditure of 10,847 Joules (averaging 1085 J per node), whereas RPLIE required 12,000 Joules. This efficiency gap widens as the network scale increases; for the 21-node configuration, EC-RPLIE consumed a total of 26,875 Joules compared to the 34,450 Joules demanded by RPLIE. The overall power consumption comparison between the different network scales and protocols is illustrated in Figure 19. While both protocols exhibit a progressive increase in consumption as the node count rises, EC-RPLIE maintains a lower energy footprint, directly enhancing individual node longevity and overall network stability.

In the most demanding scenario involving 31 nodes, the performance disparity becomes even more pronounced. EC-RPLIE recorded a total consumption of 39,196 Joules with individual node values ranging between 1030 and 1936 Joules. Although this protocol shows more irregular behavior at this scale—likely due to adaptive node characteristics or dynamic environmental variations—it remains significantly more efficient than RPLIE, which reached a total consumption of 62,000 Joules with peaks of up to 2750 Joules per node. These results validate that EC-RPLIE not only optimizes energy distribution in small-scale deployments but also provides a more scalable and robust solution for high-density networks by substantially reducing the cumulative energy burden.

### 4.3. Latency

**Figure 20:**
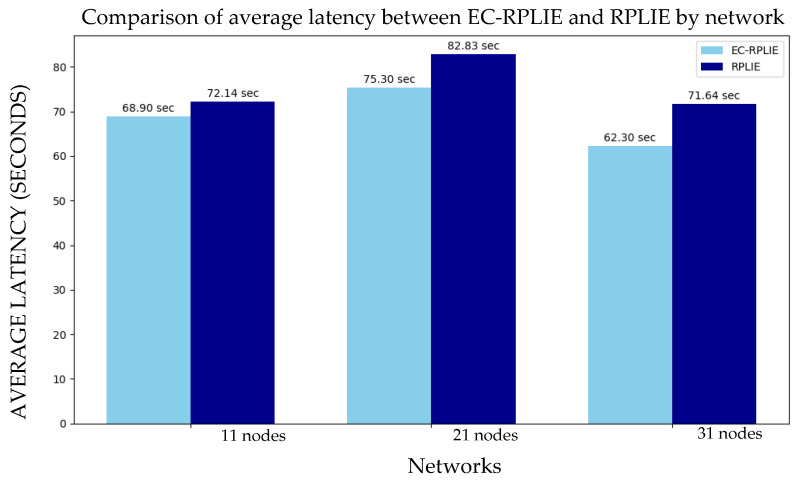
Results network latency. Source: Own elaboration.

The implementation of EC-RPLIE demonstrates a superior capacity for traffic management, consistently achieving lower average latency and reduced link jitter compared to the standard RPLIE protocol, as shown in the latency results in Figure 20. This performance gain is directly attributed to the Energy-Balanced Compression (EBC) metric, which effectively minimizes data bursts and the frequency of retransmissions. Beyond speed, this reduction in latency serves as a critical driver for energy efficiency; by minimizing the time nodes spend processing retransmissions and re-establishing parent links, EC-RPLIE optimizes power management and ensures greater stability in highly dynamic environments.

The scalability of EC-RPLIE is particularly evident in high-density deployments. In the 31-node network, the protocol shows a relative performance improvement of 12.68%, underscoring its robustness as network complexity increases. This scalability is a vital factor for Industrial Internet of Things (IIoT) applications, where reliability and sustainability are paramount. By successfully addressing these requirements, EC-RPLIE provides a resilient solution that ensures long-term network longevity and operational consistency, meeting the rigorous demands of modern industrial infrastructures.

### 4.4. PDR

**Figure 21:**
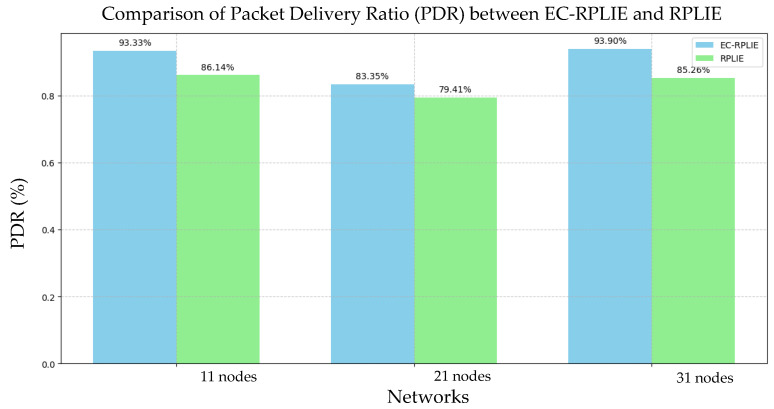
Packet Delivery Ratio results. Source: Own elaboration.

The evaluation of the Packet Delivery Ratio (PDR) reveals that EC-RPLIE maintains superior performance across all network topologies, as illustrated in Figure 21. This advantage is primarily driven by the Expected Breakage Cost (EBC) metric, which optimizes route selection and significantly mitigates packet loss. In contrast, the standard RPLIE protocol exhibits clear scalability limitations; as the network expands, its packet loss rate increases, triggering a cascade of retransmissions. These additional transmission cycles not only degrade throughput but also accelerate energy depletion, directly compromising the network’s operational lifespan.

The high PDR achieved by EC-RPLIE serves as a critical indicator of network efficiency, as it minimizes the necessity for retransmissions. This reduction in overhead leads to a dual benefit: a decrease in cumulative energy consumption and a substantial extension of individual node lifetime. Conversely, the lower PDR observed in RPLIE configurations confirms its limited efficiency in large-scale deployments, where the energy burden of retransmitting lost data becomes a bottleneck. Ultimately, the integration of the EBC metric within EC-RPLIE ensures a more resilient communication framework, providing the reliability required for high-stakes industrial environments.

## 5. Conclusions

The conducted research conclusively confirms that the Expected Breakage Cost (EBC) metric, integrated into the optimized EC-RPLIE protocol, significantly enhances energy efficiency and link stability in WSN-IIoT environments. Quantitative results demonstrated that EC-RPLIE achieves a cumulative energy reduction of up to 36.8% and improves average latency by 12.68% in high-density scenarios (31 nodes). By effectively managing medium-term fluctuations, EC-RPLIE overcomes the static limitations of traditional RPLIE, reducing unnecessary retransmissions and optimizing parent selection where energy sustainability is critical.

However, certain limitations must be acknowledged. While EC-RPLIE excels in managing medium-term fluctuations, its performance under extreme mobility or hyper-dynamic topologies (e.g., fast-moving AGVs in a warehouse) remains to be fully characterized. Additionally, the computational overhead of calculating the EBC metric on highly resource-constrained 8-bit microcontrollers could impact processing latency, a factor that was not the primary focus of this simulation-based study.

Future work will transition from simulated environments to real-world industrial testbeds to validate the EBC metric against non-deterministic electromagnetic interference. Specific research lines will include:

- Hybridization with Machine Learning: Implementing lightweight Reinforcement Learning (RL) agents to predict link breakage before it occurs, further refining the EBC calculation.
- Cross-layer Optimization: Integrating MAC-layer scheduling (such as TSCH) with EC-RPLIE to evaluate the jitter impact on real-time industrial control loops.
- Scalability Stress-Tests: Expanding the network to +100 nodes to identify the convergence time limits of the proposed objective function.

Ultimately, EC-RPLIE establishes a robust baseline for balancing energy longevity with network resilience, providing a scalable solution for the evolving demands of Industry 4.0.

## Figures and Tables

**Table 1:** Details of the simulations performed.

Parameter	Simulation 1	Simulation 2	Simulation 3
Number of nodes	11	21	31
Protocols evaluated	EC-RPLIE/RPLIE	EC-RPLIE/RPLIE	EC-RPLIE/RPLIE
Simulation time	60 min	60 min	60 min
Delivery rate (PDR)	93.33%/86.14%	83.35%/79.41%	93.90%/85.26%
Average Latency	68.90 s/72.14 s	75.30 s/82.83 s	62.30 s/71.64 s
Total energy consumption	0.000373 W/0.000405 W	0.001078 W/0.001228 W	0.001390 W/0.002122 W
Network lifetime	8.13 h/7.40 h	7.76 h/6.26 h	7.90 h/6.51 h

## Data Availability

The data presented in this study are available from the corresponding author upon reasonable request.

## Abbreviations

The following abbreviations are used in this manuscript:

RPL	Routing Protocol for Low-Power and Lossy Networks
MRHOF	Minimum Rank with Hysteresis Objective Function
PDR	Packet Delivery Ratio
EC-RPLIE	Energy Consciousness RPL for Industrial Environments
RPLIE	RPL for Indoor Environments
EBC	Expected Breakup Cost
ETX	Expected Transmission Count
WSN	Wireless Sensor Networks
IIoT	Industrial Internet of Things
IoT	Internet of Things
6LoWPAN	IPv6 over Low-Power Wireless Personal Area Networks
LLNs	Low-Power and Lossy Networks
OF	Objective Function
